# Suicidality, Psychological Inflexibility, and Emotional Resilience Among Black College Students

**DOI:** 10.3390/ijerph23060778

**Published:** 2026-06-10

**Authors:** TyWanda L. McLaurin-Jones, Shannon M. Hughley, Joi J. Wright

**Affiliations:** Department of Community and Family Medicine, Howard University College of Medicine, Howard University, 520 W Street, NW Suite 2400, Washington, DC 20059, USA; shannon.hughley@howard.edu (S.M.H.); joi.wright@howard.edu (J.J.W.)

**Keywords:** suicide, black college students, psychological inflexibility

## Abstract

**Highlights:**

**Public health relevance—How does this work relate to a public health issue?**

- Suicide is a concerning public health crisis, with suicide rates consistently rising over the past two decades, and it is a leading cause of death among young adults.
- This study examines potentially modifiable factors, specifically psychological inflexibility and emotional resilience, to identify targets for prevention and intervention strategies specific to Black college students.

**Public health significance—Why is this work of significance to public health?**

- Despite the unique social, cultural, and structural stressors faced by Black college students, limited research has examined culturally relevant risk and protective factors for suicidality among this population.
- Identifying psychological inflexibility and emotional resilience as risk or protective factors provides measurable and modifiable intervention targets, which can inform scalable mental health programs and suicide prevention efforts.

**Public health implications—What are the key implications or messages for practitioners, policymakers and/or researchers in public health?**

- Practitioners can work to identify culturally responsive ways of incorporating interventions that increase psychological flexibility, including acceptance and commitment therapy, into suicide prevention programming for Black college students.
- Policymakers and researchers can prioritize funding, research, and campus-based efforts that address culturally relevant risk factors for suicidality among Black college students.

**Abstract:**

Research examining risk and protective factors of suicidality among Black students remains limited. This study assessed the effects of psychological inflexibility and emotional resilience on suicidal behaviors among Black college students. We conducted a secondary data analysis of the 2022–2024 Healthy Minds Study. Black students (aged 18–24) who completed the suicidality matrix, psychological inflexibility (Acceptance & Action Questionnaire II) and emotional resilience (Brief Resilience Scale) measures were included in the analysis. Logistic regressions were performed to examine the effect of psychological inflexibility and resilience on suicidal ideation, plans for suicide, and suicide attempts. The students (N = 4557) represented diverse backgrounds, with 61% being African American, 12.2% African, 13.8% Caribbean, and 7% Afro-Latinx. Further, 18.7% endorsed suicidal ideation, 9.2% endorsed suicide plans, and 3.2% reported a suicidal attempt within the past 12 months. Psychological inflexibility was associated with increased risk of suicidal ideation (OR = 1.04, *p* < 0.001), suicidal plan (OR = 1.05, *p* < 0.001) and suicide attempt (OR = 1.03, *p* = 0.011). Emotional resilience was not associated with any suicidal behaviors as a protective or risk factor. The findings support previous research identifying psychological inflexibility as a suicidal risk factor. Prevention and intervention strategies may warrant a focus on promoting psychological flexibility.

## 1. Introduction

Over the past decade, the rate of suicide attempts has increased among young adults and among people with Black identity [1]. Researchers note the decline in college students’ mental health, generally seen with increasing rates of depression and anxiety, and the resulting influence on suicidal ideation among Black college students [2,3,4,5]. Yet few studies have examined suicidality among an exclusive sample of Black young adults. Among a non-college sample, Goodwill found that both young Black men and women report feeling hopeless about the future as their primary reason for considering suicide [6]. Within the college population, Black students report a significantly higher prevalence of suicidal ideation and attempts than White students [3,4,5,7,8]. One study found that Black students facing emotional exhaustion were at higher risk of suicidal ideation and attempts compared to other ethnic groups [4]. Suicidality among Black students may be less impacted by socioeconomic status. Assari found that higher income levels do not protect Black students against suicidal ideation; the risk is persistent across income levels [9]. Thus, it is imperative to investigate risk and protective factors among this vulnerable population.

To properly contextualize research regarding risk and protective factors of suicidality among Black students, it is important to understand the diversity within this population. As previous research has highlighted, race and ethnicity are complex constructs, often simplified in ways that do not reflect the heterogeneity within groups, including the Black population in the United States [10]. The Black population in the United States encompasses individuals from a wide range of historical and cultural backgrounds, including descendants of enslaved persons and naturalized citizens who have primarily emigrated from Caribbean and African countries [11,12]. Beyond nativity, heterogeneity within the Black population is further shaped by differences in socioeconomic status, gender, and phenotypic characteristics, all of which influence lived experience and social outcomes [10]. This diversity is also reflected in educational attainment as the number of Black adults earning bachelor’s and advanced degrees has increased significantly, alongside rising enrollment of Black students in colleges and universities [12,13]. Notably, factors such as discrimination, mental health stigma, cultural mistrust, and the underrepresentation of Black counseling professionals impact the mental health and service utilization of Black college and university students [14]. U.S. national data indicate that flourishing among Black students declined by 17.3% between 2013 and 2021, alongside increases in depression, anxiety, disordered eating, non-suicidal self-injury, and the prevalence of one or more mental health challenges [15].

Given the concerning suicide trend among Black college students, researchers argue for integrative theoretical approaches to understand the intersecting factors that may contribute to suicide risk [16]. Accordingly, the current investigation of suicidality is theoretically grounded in intersectionality, the cognitive–behavioral model of suicidality, and resilience theory. Intersectionality postulates that multiple social or demographic identities, like race, ethnicity, gender, and sexual orientation, interact to shape personal experiences, challenges, and opportunities [17]. Intersectionality is rooted in understanding how individual characteristics and social context (discrimination and oppression) impact health and health behaviors. When an intersectional lens is applied to suicidal behaviors, research demonstrates that gender and sexual orientation may increase vulnerability to suicide. Studies report that individuals who identify as women are at lower risk for suicide than individuals who identify as men and transgender, while other research indicates that women exhibit higher rates of repeated suicide attempts [5,18,19]. Individuals who identify as bisexual are at greater risk for suicide compared to those who identify as heterosexual, gay, or lesbian [5]. Moreover, individuals who hold multiple minoritized identities demonstrate significantly higher rates of suicidality [20].

The cognitive–behavioral model of suicidality is described as a “suicide mode” in which cognitions (suicidal belief system) and other mental processes interact with affective, behavioral, and physiological arousal components that are precipitated by predisposing vulnerabilities and stressors [21]. Predisposing vulnerabilities include psychiatric diagnosis, prior suicidal behavior, and trauma. In the general population, research consistently demonstrates that a history of suicide attempts, non-suicidal self-injury, psychiatric hospitalizations, mental health diagnoses, and trauma exposure increase suicidality [18,19,22]. For college populations, the extant literature has identified depression, trauma, non-suicidal self-injury, and substance use as risk factors for suicidality [23]. Studying predisposing vulnerabilities in the Black population could reveal nuances that may go undetected in comparative studies. For example, depression has been shown to be a stronger predictor of suicidal behaviors among Black college men than Black college women [24]. Moreover, Black students may have unique predisposing vulnerabilities that remain unaddressed in general studies. For example, Black college students identify racism as a factor that might make Black students attempt suicide [25]. Studies focused on racial minority college students demonstrate the significance of racism/discrimination in anxiety and suicidal ideation [26,27,28]. Furthermore, racism/discrimination is associated with alcohol and marijuana use, and both are independently associated with increased suicide risk [29,30,31,32,33,34,35]. Alcohol and marijuana use among Black students are linked to coping motives, and rates are increasing among this population [29,36,37,38].

Within the cognitive–behavioral model, an important mental process that remains understudied in college suicide research is psychological flexibility, a central component of acceptance and commitment therapy (ACT). Broadly, ACT is an evidence-based form of cognitive–behavioral therapy that focuses on accepting challenging thoughts and emotions and commitment to congruent values [39]. Kashdan asserts that psychological flexibility involves several “dynamic processes” that include adapting to fluctuating situational demands, reconfiguring mental resources, shifting perspectives, and balancing competing needs and desires [40]. Psychological flexibility has six dimensions (acceptance, present moment awareness, self-as-context, defusion, values, and committed action) that represent a psychologically open response to stressful experiences [41]. Higher levels of psychological flexibility are associated with lower depression and anxiety, risk factors for suicide [42,43]. Conversely, psychological inflexibility is a rigid response to stressful experiences that is characterized by experiential avoidance, lack of contact with the present moment, self-as-content, fusion, lack of contact with values, and inaction [41].

It is important to note that there are numerous critiques of the measurement of psychological flexibility [44]. Notably, psychological flexibility is commonly measured in a unidimensional manner that specifically assesses experiential avoidance, a dimension of inflexibility reflecting difficulty managing challenging internal experiences [45]. As a result of this, measures are often scored such that higher levels of experiential avoidance reflect higher levels of psychological inflexibility and are indicative of lower levels of psychological flexibility. Correspondingly, psychological inflexibility significantly predicts suicidal ideation over time among American college students [46]. Additionally, research suggests that psychological inflexibility may moderate the relationship between depression and suicidal ideation. For example, Peng and colleagues found that depression had a greater influence on suicidal ideation among students with higher experiential avoidance [47]. To expand on the current literature, it is important to assess how psychological inflexibility impacts suicidality among Black college students as this population has yet to be examined.

Researchers advocate for resilience as a focus of suicide research [48,49]. Resilience theory is a strength-based approach that focuses on positive social and individual attributes that may counteract, protect, or inoculate against risks [50,51]. Resilience is “the process and outcome of successfully adapting to difficult or challenging life experiences, especially through mental, emotional, and behavioral flexibility and adjustment to external and internal demands” [52]. Resilience is commonly analyzed using a compensatory model examining direct effects on outcomes [51]. Yet research on resilience and suicide among college students is sparse and mixed. Muyor-Rodriguz and colleagues found no relationship between resilience and suicide risk among college students in Spain [53]. However, Malak-Akgun and colleagues found that high levels of resilience reduced suicide probability among students in Turkey [54]. Similarly, Cook found that higher resilience was associated with lower suicide risk among sexual minority students [55].

Despite evidence that culture plays a critical role in shaping resilience, it remains an understudied factor in the context of suicide risk. In their meta-analysis, Rahavan and Sandanapitchai identified culture as central to the process of meaning making, the use of coping mechanisms, and values that inform resilience among those who had a traumatic experience [56]. In their review of African American suicide, Utsey and colleagues explored the role of culture in promoting resilience [57]. Their work specifically highlighted the role of culturally grounded protective factors contributing to resilience, including religious involvement, spiritual well-being, Black consciousness, and a sense of belongingness fostered through various relationships and social support networks. Given the ways in which resilience is culturally informed, it is important to explore how the role of resilience as a protective factor may differ among Black college students in the United States.

## 2. Specific Aims/Research Question

To advance our understanding of suicidality among Black college students, the current investigation examined the risk and protective effects of psychological inflexibility and emotional resilience on suicidal ideation, planning, and attempts. We hypothesize that psychological inflexibility will have a risk effect, while emotional resilience will have a protective effect.

## 3. Materials and Methods

### 3.1. Research Design and Data Source

The current investigation utilized a cross-sectional study design. Specifically, a secondary data analysis was chosen to address the specific aims. The “research question-driven approach” [58] guided the selection of the Healthy Minds Study (HMS), which specifically addresses mental health among college students. The HMS is an annual online survey that examines mental health, service utilization, and related factors among U.S. undergraduate and graduate students [59]. U.S. colleges and universities elect to enroll in the study, and students are asked to complete three modules (demographics, mental health status, and mental health help-seeking) lasting about 15 min. Students were invited to participate via email. Participants were incentivized through national and local drawings for cash, gift cards, and prizes. Per Healthy Minds Network protocol, for schools with student populations larger than 4000, a random sample of 4000 students was invited to participate, whereas the entire student body was invited to participate for smaller schools (<4000 student population). The survey was completed using Qualtrics to anonymize responses. The current investigation analyzed data collected during the 2022–2023 and the 2023–2024 academic years. Each year represented an independent sample. The HMS for 2022–2023 included 135 schools with 79,406 participants, while the HMS for 2023–2024 included 196 schools with 104,729 participants. A 9% response rate is reported for each year.

### 3.2. Study Measures

Demographics: Several participant characteristics were selected from the demographic module for descriptive and inferential analysis. Age was measured by an open-ended question, *“How old are you?”* Students were asked, *“What is your race/ethnicity?”* and selected among multiple options, including African American/Black, American Indian or Alaskan Native, Asian American/Asian, Hispanic/Latin(x), Native Hawaiian or Pacific Islander, Middle Eastern, Arab, or Arab American, or White. Race and ethnicity were further assessed by responses to *“Which group best represents your race/ethnicity?”* Students were presented with an array of groups based on their initial response to race/ethnicity. For example, if students initially selected African American/Black, they could select among the following: African, African American, Caribbean/West Indian, Afro-Latina/o/x, and other.

Gender identity was assessed via *“What is your gender identity?”* Students selected among the following choices: man, woman, transgender, genderqueer/gender non-conforming, and gender nonbinary. Students were asked about sexual orientation via *“How would you describe your sexual orientation?”* Sexual orientation responses included heterosexual, lesbian, gay, bisexual, queer, questioning, asexual, and pansexual.

Finally, academic characteristics included enrollment status, international student status, and degree program. Students were asked *“What is your enrollment status?”* and were presented with *“Full-time student”* or *“Part-time student”* as answer choices. Students were also asked, *“Are you an International student?”*, to which students responded yes or no. Degree program was assessed by *“In what degree program are you currently enrolled?”* Students chose among the following: “Associate’s”, “Bachelor’s”, “Master’s”, “JD”, “MD”, “PhD” and “Non-degree student”.

Suicidality and Self-Harm: The HMS includes three items assessing past-year suicidality in the Mental Health Status module. The suicidality matrix assesses suicidal ideation (e.g., “In the past year, did you ever seriously think about attempting suicide?), plans (e.g., “In the past year, did you make a plan for attempting suicide?”), and attempts (e.g., “In the past year, did you attempt suicide?”). Participants responded “yes” or “no” to each item to either endorse or deny suicidal behavior. Suicidality questions on the HMS align with the questions in the National Comorbidity Survey, an epidemiological survey of mental health in the United States that operationalizes diagnostic criteria [59,60]. Single-item measures of suicidality are commonly used in research and general screenings of college students.

Non-suicidal self-injury (NSSI) was assessed using a single item. Instruction for this item states, *“This question asks about ways you may have hurt yourself on purpose, without intending to kill yourself.”* This is followed by *“In the past year, have you ever done any of the following with the intent to harm yourself or manage strong emotions?”* Students were able to choose among several behaviors, including cut, burned, punched, bit, or scratched self, pulled hair, interfered with wound healing, carving into skin, and/or punched or banged object to hurt self.

Psychological Symptoms: Psychological symptoms were assessed in a number of ways. The Patient Health Questionnaire-9 (PHQ-9) was used to measure depression. Often used in the primary care setting, the PHQ-9 is a self-report brief screening tool that assess depressive symptoms during the “past two weeks” [61]. It consists of 9 items that are rated on a 4-point Likert scale, ranging from “not at all” (0) to “nearly every day” (3). Sample items include “Feeling down, depressed, or hopeless.” Scores may range from 0 to 27, with a score of ≥10 suggesting major depressive disorder. The PHQ-9 demonstrates good internal consistency (α = 0.86–0.93) among college students from diverse racial/ethnic groups [62]. An alpha of 0.90 was obtained among the current sample.

The Generalize Anxiety Disorder-7 (GAD-7) was used to measure symptoms related to anxiety. The GAD-7 is a self-report brief screening tool used in clinical practice and assesses symptoms over the “last two weeks” [63]. It consists of 7 items rated on a 4-point Likert scale, ranging from “not at all” (0) to “nearly every day” (3). Sample items include, “Feeling nervous, anxious, or on edge.” Scores may range from 0 to 21, with higher scores indicating a higher level of severity. Validation studies with college populations report high reliability (Cronbach a = 0.91–0.92) [64,65]. The GAD-7 demonstrated good internal consistency (α = 0.92) in the current sample.

Racial trauma was measured by a brief version of the Trauma Symptoms of Discrimination Scale (TSDS). The TSDS is a 21-item self- report measure that assesses the frequency of discriminatory stress [66]. Items are rated on a 4-point Likert scale ranging from never (1) to often (4). A sample item includes “Due to past experiences of racially discriminatory acts, I feel isolated and set apart from others.” Seven items from the original TSDS were included in the HMS. Flesaker and colleagues confirmed the structural validity of the brief version of TSDS and report a Cronbach’s α of 0.91 [67]. The brief version of the TSDS demonstrated good internal consistency (Cronbach’s α = 0.89) in the current sample.

Substance Use: Respondents were asked about current alcohol and marijuana use. Current alcohol use was assessed by the following question: “Over the past 2 weeks did you drink any alcohol?”, to which respondents answered yes or no. For current marijuana use, students were asked, “On the past 30 days have you used cannabis/marijuana products containing THC?”, to which students responded yes or no.

Resilience and Coping: The current investigation included two measures from the resilience and coping module. The Acceptance and Action Questionnaire-II (AAQ-II) is a 7-item self-report measure used to assess psychological inflexibility and experiential avoidance [68]. Items on the AAQ-II (e.g., “My painful experiences and memories make it difficult for me to live a life I would value”) were answered on a seven-point Likert scale ranging from 1 = never true to 7 = always true. The sum of the responses to the items was used as a total score, with lower scores reflecting greater acceptance and action and higher scores indicating higher experiential avoidance. The AAQ-II has shown good validity and reliability among many populations, including university students, with Cronbach’s α of 0.92 or greater [69,70,71]. Internal consistency in the present sample was 0.93.

The Brief Resilience Scale (BRS) is a self-report measure used to assess emotional resilience [72]. The BRS consists of a total of six items, three positively worded items (e.g., “I tend to bounce back quickly after hard times”) and three negatively worded items (e.g., “It is hard for me to snap back when something bad happens”). Items were answered on a five-point Likert-like scale ranging from 1 (strongly disagree) to 5 (strongly agree), with negatively phrased items being reverse coded. The average of the six items was used as the overall score, with higher scores reflecting higher levels of emotional resilience. The BRS has shown good validity and reliability in many populations, including among college students (Cronbach’s α = 0.84) [71]. In the present study, the BRS had good internal consistency (α = 0.82).

### 3.3. Study Sample

This investigation applied a purposive sampling strategy to the data source. Participant selection was based on two key demographics, age and race/ethnicity. Respondents who reported being between 18 and 24 years and selected Black for race/ethnicity were included in the analysis. Additionally, respondents who completed both the standard module and the resilience/coping module were included in the analysis. Participants with missing data on suicidal outcomes and scaled measures were excluded. The sampling strategy yielded 4557 participants.

### 3.4. Data Analysis

All analyses were performed using IBM SPSS Statistics 31 [73]. Descriptive statistics (frequencies and means) were calculated to assess sample demographics. Logistic regression was used to evaluate the direct effects of psychological inflexibility (AAQ-II) and emotional resilience (BRS) on suicidal behaviors using a compensatory model [51]. We aimed to predict categorical outcomes based on predictive variables while accounting for covariates. Co-variates based on the cognitive–behavioral model included depression score (PHQ-9), anxiety (GAD7), alcohol use, racial trauma score (TSDS), and past-year NSSI. Intersectionality covariates included gender and sexual orientation. Responses for gender were collapsed into three categories—(1) male, (2) female, and (3) transgender, genderqueer, or nonbinary—due to a low number of students endorsing gender identities and to increase cell sample size. Males served as the reference group in the regression. Additionally, responses for sexual orientation were collapsed into two categories—(1) heterosexual and (2) LGBTQIA+—due to low endorsement of non-heterosexual identities and to increase cell sample size. The heterosexual group served as the reference group in the regression. Those who denied alcohol use, marijuana use, and past-year NSSI served as reference groups, respectively. Assumptions were evaluated prior to analysis. Symptoms of depression and anxiety were strongly correlated in the various models for suicidal behaviors (r = 0.77–0.79); however, multicollinearity diagnostics were acceptable, with tolerance values remaining above recommended thresholds (0.34–0.37) and VIF values below problematic levels (2.70–2.98). Therefore, both variables were retained in the model. Across all predictors, multicollinearity diagnostics indicated acceptable tolerance (0.34–0.68) and VIF (1.47–2.98) values. The Box–Tidwell procedure was used to assess linearity of the logit for continuous predictors. No significant interactions between predictors and their natural log transformations were observed, indicating that the assumption of linearity of the logit was met. Examination of Cook’s distance indicated no influential outliers.

### 3.5. Sample Size Analysis

A sample size analysis was conducted using G*Power 3.1.9.7 to determine if sampling procedures were sufficient for current data analysis [74]. For chi-square tests of goodness of fit, the following parameters were selected: small effect size (0.1), alpha = 0.05, power = 0.95, and 11 degrees of freedom. The parameters yielded a minimum sample size of 2514, establishing adequacy of the current sample of 4557.

## 4. Results

### 4.1. Demographic Characteristics

Most of the sample comprised of full-time students (93.9%), with 5% identifying as international students. The sample was predominantly women (71%), heterosexual (61.2%), pursuing a bachelor’s degree (71.5%) and had a mean age of 20.24 years. The sample was ethnically diverse, with 12.2% identifying as African, 61.3% African American, 13.8% Afro-Caribbean, and 7.1% Afro-Latino (Table 1).

### 4.2. Psychological/Behavioral Characteristics

The psychological and behavioral characteristics are summarized in Table 2. Approximately 28.8% of the sample reported engaging in non-suicidal self-injury in the past year by means of cutting, burning, punching or banging, scratching, pulling hair, biting, interfering with a healing wound, carving words or symbols into skin, rubbing sharp objects into skin, or other unspecified methods. Drinking alcohol in the past two weeks was endorsed at 37.9%, while 22.7% endorsed marijuana use during the past 30 days. The mean depression score for the sample was 9.75 (SD = 6.67), reflecting mild to moderate levels of depressive symptoms. The mean anxiety score for the sample was 8.21 (SD = 6.05), reflecting mild to moderate levels of anxiety symptoms. The mean racial trauma score for the sample was 14.19 (SD = 5.89). The mean psychological inflexibility score for the sample was 23.80 (SD = 10.88). The mean emotional resilience score for the sample was 3.17 (SD = 0.76).

### 4.3. Suicidality and Suicidality Predictors

Suicidal Ideation: About 18.7% of the sample endorsed seriously thinking about suicide in the past year. The overall model for past-year suicidal ideation was statistically significant, χ^2^(11) = 1103.87, *p* < 0.001, indicating that the set of predictors reliably distinguished between participants who did and did not report suicidal ideation. The obtained pseudo-R^2^ (Nagelkerke = 0.383) indicates model fit. Psychological inflexibility was a significant predictor of suicidal ideation, whereas higher levels of psychological inflexibility were associated with increased odds of reporting suicidal ideation, [OR = 1.04, CI = (1.02–1.05), *p* < 0.001]. Resilience was not a significant predictor of suicidal ideation, [OR = 0.95, CI = (0.85–1.14), *p* = 0.786]. Among the covariates, gender, sexual orientation, marijuana use, non-suicidal self-injury, depressive symptoms, and racial trauma were significant predictors of suicidal ideation. Identifying as a woman [OR = 0.78, CI = (0.61–1.00), *p* = 0.046] was associated with decreased odds of suicidal ideation in comparison to men. Identifying as LGBTQIA [OR = 1.61, CI = (1.31–1.97), *p* < 0.001], endorsement of marijuana use [OR = 1.46, CI = (1.17–1.83), *p* < 0.001], endorsement of non-suicidal self-injury [OR = 3.53, CI = (2.90–4.30), *p* < 0.001], reporting higher levels of depressive symptoms [OR = 1.12, CI = (1.10–1.15), *p* < 0.001] and reporting higher levels of racial trauma [OR = 1.02, CI = (1.00–1.04), *p* = 0.015] were associated with increased odds of suicidal ideation. There was no association between either alcohol use or anxiety symptoms and suicidal ideation (Table 3).

Suicidal Plans: Approximately 9.2% of the sample reported planning suicide. The overall model for suicide plans was statistically significant, χ^2^(11) = 569.82, *p* < 0.001, indicating that the set of predictors reliably distinguished between participants who did and did not report having a suicide plan. The model demonstrated a good fit to the data (Nagelkerke R^2^ = 0.352). Psychological inflexibility was a significant predictor of suicide plan, whereas higher levels of psychological inflexibility were associated with increased odds of reporting having a suicide plan, [OR = 1.05, CI = (1.03–1.06), *p* < 0.001]. Resilience was not a significant predictor of having a suicide plan, [OR = 0.94, CI = (0.77–1.15), *p* = 0.556]. Among the covariates, sexual orientation, marijuana use, depressive symptoms, symptoms of anxiety, non-suicidal self-injury, and symptoms of racial trauma were significant predictors of suicide plan. Higher levels of anxiety symptoms [OR = 0.96, CI = (0.93–0.99), *p* = 0.018] were associated with decreased odds of planning suicide. Identifying as LGBTQIA [OR = 1.46, CI = (1.10–1.93), *p* = 0.009], endorsement of past 30-day marijuana use [OR = 1.52, CI = (1.13–2.04), *p* = 0.006], higher levels of depressive symptoms [OR = 1.12, CI = (1.09–1.16), *p* < 0.001], endorsement of non-suicidal self-injury [OR = 2.67, CI = (2.01–3.55), *p* < 0.001], and higher levels of racial trauma [OR = 1.03, CI = (1.01–1.05), *p* = 0.017] evidenced increased odds for planning suicide. Gender and alcohol use were not significantly associated with a suicidal plan (Table 3).

Suicide Attempts: Within the sample, the prevalence of suicide attempts was 3.2%. The overall model for suicide attempts was statistically significant, χ^2^(11) = 211.42, *p* < 0.001, indicating that the set of predictors reliably distinguished between participants who did and did not report a suicide attempt. The model demonstrated a good fit, as measured by pseudo-R^2^ (Nagelkerke R^2^ = 0.234). Psychological inflexibility was a significant predictor of suicide attempts, whereas higher levels of psychological inflexibility were associated with increased odds of reporting having a suicide attempt, [OR = 1.03, CI = (1.01–1.06), *p* = 0.011]. Resilience was not a significant predictor of a suicide attempt, [OR = 0.90, CI = (0.68–1.19), *p* = 0.456]. Among the covariates, marijuana use, depressive symptoms, and non-suicidal self-injury were significant predictors of suicide attempts. Increased odds of reporting suicidal attempts were associated with past 30-day marijuana use [OR = 1.69, CI = (1.11–2.59), *p* = 0.014], higher levels of depressive symptoms [OR = 1.06, CI = (1.02–1.11), *p* = 0.005], and endorsement of non-suicidal self-injury [OR = 3.22, CI = (2.02–5.12), *p* < 0.001]. Sexual orientation, gender, alcohol use, symptoms of anxiety, and racial trauma were not significantly related to suicidal attempts.

## 5. Discussion

The current investigation sought to further elucidate risk and protective factors of suicidality among Black college students. The investigation utilized a large sample of Black students who participated in the HMS and observed 18.7% suicidal ideation, 9.2% plans and 3.2% attempts. Other researchers analyzing the HMS report lower rates of suicidal ideation (11.5%), suicidal plans (3.9%), and suicidal attempts (0.9%) in a diverse sample of university students [75]. Lower rates of suicidal behaviors are also reported from a meta-analysis of college samples [76] and among a nationally representative sample of Black adults [77]. The study findings compare to Floyd’s observed suicidal ideation rate (19%) among Black students attending a historically Black university [31]. Research highlighting heightened suicide risk warranted further research to identify risk and protective factors specific to Black college students [8,78].

The current investigation examined suicidality through the lens of three conceptual frameworks: intersectionality, the cognitive–behavioral model, and resilience theory. Through intersectionality we examined gender identity and sexual orientation. In our sample, students identifying as women associated with decreased odds of suicidal ideation. This finding compares to Sa and colleagues’ findings that college men report higher odds of considering suicide [8]. An epidemiological study notes that Black women have one of the lowest age-adjusted rates of suicidality compared to women of other racial ethnic groups [79]. Notably, gender was not significantly associated with suicide plans or attempts, which may suggest that gender-related factors are more strongly associated with the emergence of suicidal thoughts than the progression toward suicidal behavior, which would also be consistent with ideation-to-action theories [80].

LGBTQ+ students demonstrated increased odds of reporting suicidal ideation, plans, and attempts. Our findings regarding sexual orientation are consistent with the minority stress literature, which posits that sexual and gender minorities experience health disparities because of exposure to stress based on their identity [5,18,19,20,81]. Intersectionality theory, which suggests that identities are not independent but interact to produce distinct experiences of stress, stigma, and access to support, may also inform the experiences of those who are Black and LGBTQIA [82]. Black students with minoritized sexual identities may experience overlapping systems of oppression related to racism, heterosexism, and other forms of discrimination, which may combine to inform suicide risk. Accordingly, the elevated odds of suicidal ideation observed among LGBTQ+ students in the current sample may reflect the cumulative psychological impact associated with navigating multiple minoritized identities simultaneously. Further, our findings regarding the association between racial trauma symptoms and suicidality are consistent with research that has identified a significant relationship between racial discrimination and suicidal ideation among Black college students [83,84]. This finding highlights the potential effects of racial discrimination and race-related stress exposure among Black college students.

Sexual orientation and racial trauma were associated with suicidal ideation and planning but not suicide attempts, which may suggest that identity-related and race-related stressors are particularly relevant to the development and escalation of suicidal thoughts, while other factors may play a larger role in progression to suicidal behaviors. This is consistent with ideation-to-action theories of suicide, which suggest that there are unique factors that contribute to suicidal ideation and differ from those that contribute to suicide attempts [80].

Within the cognitive model of suicide framework, we examined several psychological factors. Higher levels of depressive symptoms, endorsement of past 30-day marijuana use, and past-year NSSI predicted suicidal ideation, suicide plans, and suicide attempts in the current sample, highlighting these factors as potentially important markers of suicide risk among Black college students. The association between depressive symptoms and suicidality in the current sample parallels findings from numerous studies of Black college students and young adults identifying depression as a correlate of suicidality [24,85,86]. Similarly, our findings regarding marijuana are consistent with previous studies that have identified associations between cannabis use and suicidal ideation, plans, and attempts in several populations [87,88]. Notably, Floyd found that African American college students with a history of early-onset marijuana use were more likely to report suicidal ideation than those who denied marijuana use [31].

Previous studies have demonstrated that NSSI is associated with greater risk of suicide attempts among students with suicidal ideation and those not reporting suicidal ideation, which is consistent with our findings regarding the associations between NSSI and suicidal behaviors [89,90,91,92]. Notably, the past-year prevalence of NSSI in our sample of Black college-aged students (28.8%) exceeded the rates reported in undergraduate students more generally (7%), among Black college students attending a predominantly White institution (10%) and among students attending a historically Black college and university (16%) [89,90,91,92]. Previous research has identified gender differences in NSSI prevalence among Black university students, with 22.5% of men endorsing NSSI compared to 12.2% of women [91]. Although not examined, gender differences may be underlying the elevated rates of NSSI within our study.

As noted, several variables demonstrated differential associations across suicidal ideation, planning, and attempts, suggesting that these suicidal behaviors may represent related but distinct processes rather than a single continuum. For example, racial trauma was associated with suicidal ideation and planning but did not significantly predict suicide attempts. In contrast, NSSI emerged as a predictor across all suicidal behaviors. These findings align with ideation-to-action theories and may be indicative of different factors contributing to suicidal thoughts compared to those involved in the progression to suicide attempts [80]. Additional longitudinal research is needed to clarify how these mechanisms differentially influence suicidality among Black college students.

Notably, higher levels of anxiety symptoms were associated with lower odds of endorsing suicidal plans, although anxiety symptoms were not associated with suicidal ideation or attempts. This finding contrasts with research identifying anxiety as a risk factor for suicidality [93] Previous research has also identified an indirect association between racial and ethnic discrimination and suicidal thoughts through generalized anxiety, but not race-based anxiety or social anxiety, among individuals of various minoritized racial ethnic backgrounds, which suggests that certain types of anxiety may have differential impacts on suicidality [94].

Our hypothesis that psychological inflexibility increases risk of suicidality was supported across all three measures of suicidality: ideation, plan, and attempt. Prior studies report similar findings in a cross-sectional and longitudinal analysis of psychological inflexibility predicting suicidal ideation among students attending a PWI and psychological inflexibility’s association with higher suicide risk among students attending a Tribal college [46,95,96]. While our study demonstrated a significant association between inflexibility and suicide attempts, Polanco-Roman found no relationship between inflexibility and suicide attempts among college students [94]. The mean psychological inflexibility score observed in the current sample parallels the mean reported in a diverse sample of undergraduate students [97].

Drawing from resilience theory, our hypothesis that resilience would exert a protective effect on suicidal behaviors was not supported. These findings are consistent with research demonstrating that resilience did not distinguish between students reporting suicidal ideation or suicidal attempts [98] and contrast with other studies demonstrating that resilience is associated with lower suicide risk [96,99]. The level of emotional resilience observed in the current sample is comparable to the level observed among racial/ethnic minority college students [100].

Several limitations of the present study should be acknowledged when interpreting the findings. First, a cross-sectional research design cannot establish causality. Self-report survey data is subject to the possibility of self-selection bias, a well-documented limitation of survey-based research, which should be considered when interpreting the findings of the present study. Notably, previous research has suggested that college students who self-select to participate in health-related research have more favorable health outcomes than their peers [101]. Therefore, we must consider this a possibility for students who opted to participate in the HMS and take this into consideration when attempting to generalize the findings of the current study.

Second, the analytic approach for gender identity and sexual orientation was constrained by insufficient representation within certain categories, necessitating the collapse of groups into broader classifications (i.e., transgender, genderqueer, nonbinary, and LGBTQIA+). Collapsing categories may increase the risk of false positives, may impact overgeneralization, or may have obscured meaningful subgroup differences and reduced the specificity and nuance of the findings. As a result, our findings regarding gender and suicidality should be interpreted cautiously and may reflect methodological and contextual factors. Several previous studies exhibiting stronger associations between gender and suicidality have utilized gender minority populations specifically, which may account for the discrepancies in our findings compared to previous research [102].

Thirdly, conducting a secondary data analysis of an existing dataset constrained measurement selection. Using national surveys with single items to assess suicidal behaviors may lead to misclassification and may have impacted the rates of suicidality observed in the sample [103,104]. A validated multi-item instrument, such as the Columbia-Suicide Severity Scale, is more commonly used in research and may more accurately assess suicide [105]. Likewise, the AAQ-II, as a measure of psychological inflexibility (experiential avoidance), has received criticism. Psychometric studies report concerns about discriminant validity, noting that the AAQ-II measures psychological distress [106,107]. The Multidimensional Psychological Flexibility Inventory is a validated instrument that can measure both flexibility and inflexibility [108].

Despite the noted limitations, the present study has several notable strengths and makes meaningful contributions to the literature on suicidality among college students [6,25,31,57,77]. Importantly, the study addresses a significant gap in the existing research by centering on Black college-aged students, a population that has historically been underrepresented in suicidality research. Given the recent concerning trends in suicidality among Black college students identified in the current study and previous research, it is particularly important for research to assess risk and protective factors for suicidality among this population to inform suicide prevention interventions [78].

Additionally, the sample was drawn from diverse Black college-aged students from a range of institutions and geographic regions across the United States. The institutional and regional diversity further supports the generalizability of the findings to a broader range of Black college students in the United States.

The study also examined theoretically and clinically relevant psychological constructs, specifically psychological inflexibility/experiential avoidance and emotional resilience, which previous studies have identified as relevant for suicide prevention and intervention efforts [49,109]. The current study has helped to elucidate the roles of psychological inflexibility/experiential avoidance and emotional resilience in relation to suicidal behaviors among Black college-aged students. The findings of this study may inform culturally responsive suicide prevention interventions specifically aiming to decrease psychological inflexibility.

## 6. Future Research

Future research can build on the findings of the present study by prioritizing the development, adaptation, and evaluation of suicide prevention interventions tailored to Black college students. Although the majority of the sample consisted of Black American students from the United States, the sample was not culturally homogeneous and included college students who identified as Caribbean, African, and Afro-Latinx. Consequently, culturally specific or contextual factors that may shape the participants’ experiences were not explicitly captured in the analyses, limiting the interpretability and generalizability of the findings across diverse Black populations. Prior research has identified variability in suicidality across Black ethnic groups in the general Black adult population [110]. Similarly, suicidality varies between domestic and international students [111]. Future studies should examine ethnic and cultural subgroup differences to inform culturally responsive interventions that account for the heterogeneity within Black student populations. Such disaggregation is essential for identifying subgroup-specific risk and protective factors that can be specifically targeted in suicide prevention efforts.

Similarly, future intervention development efforts should also adopt intersectional frameworks that explicitly consider gender identity and sexual orientation. Prior research has demonstrated intersectional differences in the prevalence of suicidal ideation based on gender identity and sexual orientation, with bisexual women reporting higher rates of suicidal ideation than lesbian women and lesbian, gay, and bisexual adults exhibiting higher rates of suicidality than heterosexual adults [112]. Larger and more diverse samples are needed to support nuanced analyses of Black transgender, genderqueer, nonbinary, and LGBTQIA+ students, who may face compounded stressors related to marginalization, as posited by the minority stress model [113]. Understanding how psychological inflexibility/experiential avoidance and emotional resilience function across these intersecting identities can inform the design of suicide prevention interventions that are both affirming of and responsive to the unique needs of these groups.

In addition, future research can evaluate the efficacy of interventions that directly target psychological inflexibility as a risk factor for suicidal behaviors among Black college students. Longitudinal and experimental designs, including randomized controlled trials, are needed to determine whether interventions aimed at reducing psychological inflexibility/experiential avoidance contribute to reduced suicidality over time. Such work would strengthen causal inference and support the translation of research findings into tailored evidence-based suicide prevention programming and interventions for Black college students.

Lastly, future research should also examine alternative conceptualizations of resilience beyond emotional resilience alone. Cognitive resilience is defined by Staal and colleagues as “the capacity to overcome the negative effects of setbacks and associated stress on cognitive function or performance” [114]. Cognitive flexibility may represent a more salient protective factor against suicidality among Black college students given the ways in which culture informs resilience among African Americans [57,114]. Based on the observed relationship between psychological inflexibility and suicidal behaviors in the current study, constructs emphasizing cognitive adaptability may be particularly relevant for intervention development. Examining multiple dimensions of resilience may help to clarify which forms of resilience are most protective against suicidality within diverse Black student populations. Future studies may also consider examining indirect effects of resilience. Studies demonstrate that resilience may moderate the relationship between psychological symptoms (depression and anxiety) and suicidal behaviors [115].

## 7. Conclusions

In conclusion, the present study contributes to the growing body of literature examining suicidality among Black college students by examining risk and protective factors. The elevated rates of suicidal behaviors observed in this sample underscore the urgent need for culturally responsive suicide prevention efforts tailored to Black college-aged populations. We identified experiential avoidance, a dimension of psychological inflexibility, as a significant risk factor for suicidal ideation, planning, and attempts. Although emotional resilience did not demonstrate a direct protective effect against suicidality, the findings suggest that resilience may operate through more complex or indirect pathways that warrant further investigation. Importantly, the study highlights the need to account for the heterogeneity and intersecting identities within Black student populations, including differences related to ethnicity, gender identity, and sexual orientation, when examining suicide risk and protective factors. Collectively, these findings support continued research aimed at clarifying the culturally relevant mechanisms underlying suicidality and informing the development of targeted evidence-based interventions to reduce suicide risk and promote mental health among Black college students.

## Figures and Tables

**Table 1:** Black college students’ demographic characteristics (N = 4557).

Demographic Characteristic	Descriptive Statistics
Age (Mean ± SD)	20.24 ± 1.77
Black Ethnicity [n (%)]	
African	558 (12.2%)
African American	2791 (61.3%)
Caribbean	630 (13.8%)
Afro-Latinx	323 (7.1%)
Not Reported	255 (5.6%)
Gender [n (%)]	
Male	1007 (22.1%)
Female	3236 (71.0%)
Trans, Queer, or Nonbinary	260 (5.7%)
Not Reported	54 (1.2%)
Sexual Orientation [n (%)]	
Heterosexual	2788 (61.2%)
LGBTQIA+	1380 (30.3%)
Not Reported	389 (8.5%)
Degree Program [n (%)]	
Associate’s	765 (16.8%)
Bachelor’s	3259 (71.5%)
Master’s	283 (6.2%)
Doctorate (PhD, MD, or JD)	121 (2.7%)
Non-Degree	46 (1.0%)
Not Reported	83 (1.8%)
Nationality [n (%)]	
United States	4313 (94.6%)
International	244 (5.4%)
Enrollment Status [n (%)]	
Full-Time Student	4281 (93.9%)
Part-Time Student	240 (5.3%)
Not Reported	36 (0.8%)

**Table 2:** Black college students’ psychological and behavioral characteristics (N = 4557).

Characteristic	% or M (SD)
Marijuana use	22.7%
Alcohol use	37.9%
Non-suicidal self-injury	28.8%
Suicidal ideation	18.7%
Suicide plan	9.2%
Suicide attempt	3.2%
PHQ-9	9.75 (6.67)
GAD-7	8.21 (6.05)
TSDS	14.19 (5.89)
AAQ-2	23.80 (10.88)
BRS	3.17 (0.76)

M = mean. SD = standard deviation.

**Table 3:** Predictors of suicidal behaviors among Black college students (N = 4557).

Predictor	Suicidal Ideation				Suicidal Plan				Suicidal Attempt			
	OR	95% CI		*p*	OR	95% CI		*p*	OR	95% CI		*p*
		LL	UL			LL	UL			LL	UL	
Gender												
Women	0.78	0.61	1.00	0.046	0.77	0.55	1.10	0.148	0.75	0.45	1.24	0.265
Trans/Queer/Nonbinary	0.69	0.45	1.05	0.083	0.96	0.57	1.64	0.886	0.82	0.39	1.74	0.611
Sexual Orientation	1.61	1.31	1.97	<0.001	1.46	1.10	1.93	0.009	1.00	0.66	1.52	0.986
Alcohol	1.11	0.90	1.36	0.345	1.13	0.85	1.50	0.404	0.96	0.63	1.45	0.840
Marijuana	1.46	1.17	1.83	<0.001	1.52	1.13	2.04	0.006	1.69	1.11	2.59	0.014
Depression	1.12	1.10	1.15	<0.001	1.12	1.19	1.16	<0.001	1.06	1.02	1.11	0.005
Anxiety	0.98	0.96	1.01	0.110	0.96	0.93	0.99	0.018	1.01	0.96	1.07	0.608
Non-Suicidal Self-Injury	3.53	2.90	4.30	<0.001	2.67	2.01	3.55	<0.001	3.22	2.02	5.12	<0.001
Trauma	1.02	1.00	1.04	0.015	1.03	1.01	1.05	0.017	1.03	0.99	1.07	0.100
Inflexibility	1.04	1.02	1.05	<0.001	1.05	1.05	1.06	<0.001	1.03	1.01	1.06	0.011
Emotional Resilience	0.95	0.85	1.14	0.786	0.94	0.77	1.15	0.556	0.90	0.68	1.19	0.456

OR = odds ratio. CI = confidence interval. LL = lower limit. UL = upper limit.

## Data Availability

Data acquired and used for this study may be requested from the Health Minds Network at https://healthymindsnetwork.org/.

## References

[1] Bommersbach T.J., Rosenheck R.A., Rhee T.G. (2022). National trends of mental health care among US adults who attempted suicide in the past 12 months. JAMA Psychiatry.

[2] Duffy M.E., Twenge J.M., Joiner T.E. (2019). Trends in mood and anxiety symptoms and suicide-related outcomes among U.S. undergraduates, 2007–2018: Evidence from two national surveys. J. Adolesc. Health.

[3] Chen J.A., Stevens C., Wong S.H.M., Liu C.H. (2019). Psychiatric symptoms and diagnoses among U.S. college students: A comparison by race and ethnicity. Psychiatr. Serv..

[4] Lin H.-C., Li M., Stevens C., Pinder-Amaker S., Chen J.A., Liu C.H. (2023). Self-harm and suicidality in US college students: Associations with emotional exhaustion versus multiple psychiatric symptoms. J. Am. Coll. Health.

[5] Liu C.H., Stevens C., Wong S.H.M., Yasui M., Chen J.A. (2019). The prevalence and predictors of mental health diagnoses and suicide among U.S. college students: Implications for addressing disparities in service use. Depress. Anxiety.

[6] Goodwill J.R. (2024). Reasons for suicide in Black young adults: A latent class analysis. J. Racial Ethn. Health Disparities.

[7] Kodish T., Lau A.S., Gong-Guy E., Congdon E., Arnaudova I., Schmidt M., Shoemaker L., Craske M.G. (2022). Enhancing racial/ethnic equity in college student mental health through innovative screening and treatment. Adm. Policy Ment. Health.

[8] Sa J., Choe C.S., Cho C.B., Chaput J.P., Lee J., Hwang S. (2020). Sex and racial/ethnic differences in suicidal consideration and suicide attempts among US college students, 2011–2015. Am. J. Health Behav..

[9] Assari S. (2019). Subjective financial status and suicidal ideation among American college students: Racial differences. Arch. Intern. Med..

[10] Celious A., Oyserman D. (2001). Race from the inside: An emerging heterogeneous race model. J. Soc. Issues.

[11] Agyemang C., Bhopal R., Bruijnzeels M. (2005). Negro, Black, Black African, African Caribbean, African American or what? Labelling African origin populations in the health arena in the 21st century. J. Epidemiol. Community Health.

[12] Pew Research Center Key Findings About Black America in 2019. https://www.pewresearch.org/short-reads/2021/03/25/key-findings-about-black-america/.

[13] United States Census Bureau Black igh School Attainment Early on par with National Average. https://www.census.gov/library/stories/2020/06/black-high-school-attainment-nearly-on-par-with-national-average.html.

[14] Leath S., Jones M. (2022). Racial climate and mental health service utilization among Black college students at diverse institutions. Curr. J. Divers. Scholarsh. Soc. Change.

[15] Lipson S.K., Zhou S., Abelson S., Heinze J., Jirsa M., Morigney J., Patterson A., Singh M., Eisenberg D. (2022). Trends in college student mental health and help-seeking by race/ethnicity: Findings from the national healthy minds study, 2013–2021. J. Affect. Disord..

[16] Opara I., Assan M.A., Pierre K., Gunn J.F., Metzger I., Hamilton J., Arugu E. (2022). Suicide among black children: An integrated model of the interpersonal-psychological theory of suicide and intersectionality theory for researchers and clinicians. Focus (Am. Psychiatr. Publ.).

[17] Crenshaw K. (1991). Mapping the margins: Identity politics, intersectionality, and violence against women. Stanf. Law Rev..

[18] Park C.G.K., Lee J.W., Lee S.Y., Moon J., Jeon D., Shim S., Cho S., Kim S.G., Lee J., Paik J. (2020). Suicide risk factors across suicidal ideators, single suicide attempters, and multiple suicide attempters. J. Psychiatr. Res..

[19] Steele I.H., Thrower N., Noroian P., Saleh F.M. (2018). Understanding suicide across the lifespan: A United States perspective of suicide risk factors, assessment, and management. J. Forensic Sci..

[20] Forrest L.N., Beccia A.L., Exten C., Gehman S., Ansell E.B. (2023). Intersectional prevalence of suicide ideation, plan, and attempt based on gender, sexual orientation, race and ethnicity, and rurality. JAMA Psychiatry.

[21] Rudd M.D. (2000). The suicidal mode: A cognitive-behavioral model of suicidality. Suicide Life-Threat. Behav..

[22] Owusu-Ansah F.E., Addae A.A., Peasah B.O., Asante K.O., Osafo J. (2020). Suicide among university students: Prevalence, risks and protective factors. Health Psychol. Behav. Med..

[23] Zhao M., Xin Y., Bai X., Zhang S., Liu H., Xu W., Duan W., Jin Q., Chen Y., Luo Y. (2025). Risk factors for suicidality among college students: A systematic review and meta-analysis. J. Affect. Disord..

[24] Wang M.C., Lightsey O.R., Tran K.K., Bonaparte T.S. (2013). Examining suicide protective factors among black college students. Death Stud..

[25] Borum V. (2014). African Americans’ perceived sociocultural determinants of suicide: Afrocentric implications for public health inequalities. Soc. Work Public Health.

[26] Polanco-Roman L., Hollingsworth D.W., Liang C., Oduro N., Anglin D.M. (2022). Racial/ethnic discrimination, anxiety, and suicidal thoughts among ethnoracially minoritized college students. Am. J. Orthopsychiatry.

[27] Szlyk H., Motley R., Joe S., Nonas-Barnes L., Azasu E. (2022). An examination of suicidal behavior among black college students with exposure to police violence. Soc. Work.

[28] Chao R.C., Mallinckrodt B., Wei M. (2012). Co-occurring presenting problems in African American college clients reporting racial discrimination distress. Prof. Psychol. Res. Pract..

[29] Buckner J.D., Sullivan J.M., Thomas K.L., Shepherd J.M., Zvolensky M.J. (2024). Racism and alcohol-related problems among Black adults: The role of negative emotionality to experiencing racism. J. Subst. Use Addict. Treat..

[30] Daneshmend A.Z.B., Stewart J., Jarkas D.A., Franklyn S.I., Gabrys R.L., Patterson Z.R., Abizaid A., Hellemans K.G.C., McQuaid R.J. (2022). Examining risk factors in the cannabis–suicide link: Considering trauma and impulsivity among university students. Int. J. Environ. Res. Public Health.

[31] Floyd L.J. (2025). Early onset marijuana use and suicidal ideation among African American college students. J. Ethn. Subst. Abus..

[32] Lamis D.A., Malone P.S., Jahn D.R. (2014). Alcohol use and suicide proneness in college students: A proposed model. Ment. Health Sub. Use.

[33] Pittman D.M., Kaur P. (2018). Examining the role of racism in the risky alcohol use behaviors of black female college students. J. Am. Coll. Health.

[34] Trangenstein P.J., Yeh J.C., Sparks A., Arria A.M., Greenfield T.K., Jernigan D.H. (2025). Alcohol’s collateral damage: Harms from others’ drinking are linked to academic and mental health challenges among U.S. college students. J. Stud. Alcohol Drugs.

[35] Vidal C., Alvarez P., Hammond C.J., Lilly F.R.W. (2023). Cannabis use associations with adverse psychosocial functioning among North American college students. Subst. Use Misuse.

[36] Hai A.H., Carey K.B., Vaughn M.G., Lee C.S., Franklin C., Salas-Wright C.P. (2022). Simultaneous alcohol and marijuana use among college students in the United States, 2006–2019. Addict. Behav. Rep..

[37] O’Hara R.E., Boynton M.H., Scott D.M., Armeli S., Tennen H., Williams C., Covault J. (2014). Drinking to cope among African American college students: An assessment of episode-specific motives. Psychol. Addict. Behav..

[38] Pittman D.M., Brooks J.J., Kaur P., Obasi E.M. (2019). The cost of minority stress: Risky alcohol use and coping-motivated drinking behavior in African American college students. J. Ethn. Subst. Abus..

[39] Anusuya S.P., Gayatridevi S. (2025). Acceptance and commitment therapy and psychological well-being: A narrative review. Cureus.

[40] Kashdan T.B., Rottenberg J. (2010). Psychological flexibility as a fundamental aspect of health. Clin. Psychol. Rev..

[41] Hayes S.C., Strosahl K.D., Wilson K.G. (2012). Acceptance and Commitment Therapy: The Process and Practice of Mindful Change.

[42] Browning M.E., Lloyd-Richardson E.E., Trisal A.V., Satterfield S. (2024). Anxiety, OCD, psychological flexibility, and depression: A replication with racially minoritized university students. Psychol. Rep..

[43] Landi G., Pakenham K.I., Crocetti E., Tossani E., Grandi S. (2022). The trajectories of anxiety and depression during the COVID-19 pandemic and the protective role of psychological flexibility: A four-wave longitudinal study. J. Affect. Disord..

[44] Doorley J.D., Goodman F.R., Kelso K.C., Kashdan T.D. (2020). Psychological flexibility: What we know, what we do not know, and what we think we know. Soc. Pers. Psychol. Compass.

[45] Hsu T., Hoffman L., Thomas E.B.K. (2023). Confirmatory measurement modeling and longitudinal invariance of the CompACT-15: A short-form assessment of psychological flexibility. Psychol. Assess..

[46] Krafft J., Hicks E.T., Mack S.A., Levin M.E. (2019). Psychological inflexibility predicts suicidality over time in college students. Suicide Life Threat. Behav..

[47] Peng B., Hu N., Guan L., Chen C., Chen Z., Yu H. (2023). Family functioning and suicidal ideation in college students: A moderated mediation model of depression and acceptance. Front. Public Health.

[48] Robinson W.L., Whipple C.R., Keenan K., Flack C.E., Wingate L. (2022). Suicide in African American Adolescents: Understanding Risk by Studying Resilience. Annu. Rev. Clin. Psychol..

[49] Sher L. (2019). Resilience as a focus of suicide Research and prevention. Acta Psychiatr. Scand..

[50] Fergus S., Zimmerman M.A. (2005). Adolescent resilience: A framework for understanding healthy development in the face of risk. Annu. Rev. Public Health.

[51] Zimmerman M.A. (2013). Resiliency theory: A strengths-based approach to research and practice for adolescent health. Health Educ. Behav..

[52] American Psychological Association (2018). Dictionary of Psychology.

[53] Muyor-Rodríguez J., Caravaca-Sánchez F., Fernández-Prados J.S. (2021). COVID-19 fear, resilience, social support, anxiety, and suicide among college students in Spain. Int. J. Environ. Res. Public Health.

[54] Malak-Akgün B., Üzar-Özçetin Y.S., Aydin A. (2022). Association between resilience, self-esteem and suicide probability among university students in Turkey. Perspect. Psychiatr. Care.

[55] Cook K. (2025). Suicide risk and protective factors for sexual minority college students: A latent class analysis. OMEGA-J. Death Dying.

[56] Raghavan S., Sandanapitchai P. (2004). The relationship between cultural variables and resilience to psychological trauma: A systematic review of the literature. Traumatology.

[57] Utsey S.O., Hook J.N., Stanard P. (2007). A re-examination of cultural factors that mitigate risk and promote resilience in relation to African American Suicide: A review of the literature and recommendations for future research. Death Stud..

[58] Cheng H.G., Phillips M.R. (2014). Secondary analysis of existing data: Opportunities and implementation. Shanghai Arch. Psychiatry.

[59] Healthy Minds Network (2024). Healthy Minds Study Among Colleges and Universities, 2022–2024 [Data Set]. Healthy Minds Network, University of Michigan, University of California Los Angeles, Boston University, and Wayne State University. https://healthymindsnetwork.org/research/data-for-researchers.

[60] Kessler R.C., Merikangas K.R. (2004). The national comorbidity survey replication (NCS-R): Background and aims. Int. J. Methods Psychiatr. Res..

[61] Kroenke K., Spitzer R.L., Williams J.B. (2001). The PHQ-9: Validity of a brief depression severity measure. J. Gen. Intern. Med..

[62] Keum B.T., Miller M.J., Inkelas K.K. (2018). Testing the factor structure and measurement invariance of the PHQ-9 across racially diverse U.S. college students. Psychol. Assess..

[63] Spitzer R.L., Kroenke K., Williams J.B., Löwe B. (2006). A brief measure for assessing generalized anxiety disorder: The GAD-7. Arch. Intern. Med..

[64] Byrd-Bredbenner C., Eck K., Quick V. (2021). GAD-7, GAD-2, and GAD-mini: Psychometric properties and norms of university students in the United States. Gen. Hosp. Psychiatry.

[65] White A.E., Karr J.E. (2025). Psychometric properties of the GAD-7 among college students: Reliability, validity, factor structure, and measurement invariance. Transl. Issues Psychol. Sci..

[66] Williams M.T., Printz D.M.B., DeLapp R.C.T. (2018). Assessing racial trauma with the trauma symptoms of discrimination scale. Psychol. Violence.

[67] Flesaker M., Schneider B., Patterson A., Rosellini A.J., Lipson S.K., Gradus J.L. (2025). Co-occurrence of racism-based trauma symptoms and depressive symptoms among college students of color. Psychol. Trauma.

[68] Bond F.W., Hayes S.C., Baer R.A., Carpenter K.M., Guenole N., Orcutt H.K., Waltz T., Zettle R.D. (2011). Preliminary psychometric properties of the acceptance and action questionnaire II: A revised measure of psychological inflexibility and experiential avoidance. Behav. Ther..

[69] Ganson K.T., O’Connor J., Nagata J.M., Lipson S.K. (2023). Association between psychological flexibility and physical violence perpetration in college student populations: Results from the National Healthy Minds Study. J. Am. Coll. Health.

[70] Hannan S.M., Orcutt H.K. (2020). Emotion regulation in undergraduate students with posttraumatic stress symptoms: A multimethod study. Psychol. Trauma.

[71] Khalil Z., Mason T.B., Smith K. (2025). Examining racial-ethnic and gender differences in the associations between resilience, psychological inflexibility, and eating disorders. J. Am. Coll. Health.

[72] Smith B.W., Dalen J., Wiggins K., Tooley E., Christopher P., Bernard J. (2008). The Brief Resilience Scale: Assessing the ability to bounce back. Int. J. Behav. Med..

[73] IBM Corp (2025). IBM SPSS Statistics for Windows.

[74] Faul F., Erdfelder E., Buchner A., Lang A.-G. (2009). Statistical power analyses using G*Power 3.1: Tests for correlation and regression analyses. Behav. Res. Methods.

[75] Ning K., Yan C., Zhang Y., Chen S. (2022). Regular exercise with suicide ideation, suicide plan and suicide attempt in university students: Data from the Health Minds Survey 2018–2019. Int. J. Environ. Res. Public Health.

[76] Mortier P., Cuijpers P., Kiekens G., Auerbach R.P., Demyttenaere K., Green J.G., Kessler R.C., Nock M.K., Bruffaerts R. (2018). The prevalence of suicidal thoughts and behaviours among college students: A meta-analysis. Psychol. Med..

[77] Yockey A., Hoopsick R., Clary K., Devier E., Amis A., Silva R., Brantley L., Gobin R. (2025). Suicidal ideation, planning, and attempts among Black/African American adults: Differences by sexual orientation 2023, USA. Health Behav. Res..

[78] Busby D.R., Zheng K., Eisenberg D., Albucher R.C., Favorite T., Coryell W., Pistorello J., King C.A. (2021). Black college students at elevated risk for suicide: Barriers to mental health service utilization. J. Am. Coll. Health.

[79] Price J.H., Foh E.P. (2024). Descriptive Epidemiology of female suicides by race and ethnicity. J. Community Health.

[80] Bayliss L.T., Christensen S., Lamont-Mills A., du Plessis C. (2022). Suicide capability within the ideation-to-action framework: A systematic scoping review. PLoS ONE.

[81] Frost D.M., Meyer I.H. (2023). Minority stress theory: Application, critique, and continued relevance. Curr. Opin. Psychol..

[82] Phoenix A., Pattynama P. (2006). Intersectionality. Eur. J. Women’s Stud..

[83] Goodwill J.R., Taylor R.J., Watkins D.C. (2021). Everyday discrimination, depressive symptoms, and suicide ideation among African American men. Arch. Suicide Res..

[84] Rule A., Davis Molock S., Job S., Siff L. (2026). Perceived racial discrimination as a risk factor for suicidal ideation in Black college students. J. Coll. Stud. Ment. Health.

[85] Brooks J.R., Madubata I.J., Jewell R.D., Ortiz D.A., Walker R. (2023). Depression and suicide ideation: The role of self-acceptance for Black young adults. J. Black Psychol..

[86] Morrison K.S., Hopkins R. (2019). Cultural identity, africultural coping strategies, and depression as predictors of suicidal ideations and attempts among African American female college students. J. Black Psychol..

[87] Shamabadi A., Ahmadzade A., Pirahesh K., Hasanzadeh A., Asadigandomani H. (2023). Suicidality risk after using cannabis and cannabinoids: An umbrella review. Dialogues Clin. Neurosci..

[88] Gobbi G., Atkin T., Zytynski T., Wang S., Askari S., Boruff J., Ware M., Marmorstein N., Cipriani A., Dendukuri N. (2019). Association of cannabis use in adolescence and risk of depression, anxiety, and suicidality in young adulthood: A systematic review and meta-analysis. JAMA Psychiatry.

[89] Bakken V., Skokauskas N., Sund A.M., Kaasbøll J. (2025). Protective factors for suicidality: A qualitative follow-up of the Youth and Mental Health Study cohort. BMC Public Health.

[90] Chesin M.S., Moster A.N., Jeglic E.L. (2016). Non-suicidal self-injury among ethnically and racially diverse emerging adults: Do factors unique to the minority experience matter?. Curr. Psychol..

[91] Davis L.T., Weiss N.H., Tull M.T., Gratz K.L. (2017). The relation of protective factors to deliberate self-harm among African American adults: Moderating roles of gender and sexual orientation identity. J. Ment. Health.

[92] McCollum D.C., Smathers S.E., Sullivan T., Jowaheer Y., Mereish E.H. (2025). Associations among intimate partner violence, suicidal ideation, suicide behaviors, non-suicidal self-injury, and psychological well-being in Black American emerging adults. Suicide Life Threat. Behav..

[93] Bentley K.H., Franklin J.C., Ribeiro J.D., Kleiman E.M., Fox K.R., Nock M.K. (2016). Anxiety and its disorders as risk factors for suicidal thoughts and behaviors: A meta-analytic review. Clin. Psychol. Rev..

[94] Polanco-Roman L., Jurska J., Quiñones V., Miranda R. (2015). Brooding, reflection, and distraction: Relation to non-suicidal self-injury versus suicide attempts. Arch. Suicide Res..

[95] Chou W.P., Yen C.F., Liu T.L. (2018). Predicting effects of psychological inflexibility/experiential avoidance and stress coping strategies for internet addiction, significant depression, and suicidality in college students: A prospective study. Int. J. Environ. Res. Public Health.

[96] Parker M., Duran B., Rhew I., Magarati M., Larimer M., Donovan D. (2021). Risk and protective factors associated with moderate and acute suicidal ideation among a national sample of tribal college and university students 2015–2016. J. Rural Health.

[97] Laman-Maharg B., Valentiner D.P., Szöllös S., Mounts N.S. (2025). Experiential avoidance, post-traumatic stress symptoms, and academic impairment. Psychol. Rep..

[98] Aizpurua E., Caravaca-Sánchez F., Taliaferro L.A. (2022). Suicidality among college students in Spain: Prevalence and associations with substance use, social support, and resilience. Death Stud..

[99] Luceño-Moreno L., Vázquez-Estévez D., Martín-García J., Talavera-Velasco B. (2025). Factors associated with suicidal ideation in college students of health sciences. Depress. Anxiety.

[100] Warren J.M., Hale R.W. (2020). Predicting grit and resilience: Exploring college students’ academic rational beliefs. J. Coll. Couns..

[101] Ludy M.J., Crum A.P., Young C.A., Morgan A.L., Tucker R.M. (2018). First-year university students who self-select into health studies have more desirable health measures and behaviors at baseline, but experience similar changes compared to non-self-selected students. Nutrients.

[102] Miranda-Mendizabal A., Castellví P., Parés-Badell O., Alayo I., Almenara J., Alonso I., Blasco M.J., Cebrià A., Gabilondo A., Gili M. (2019). Gender differences in suicidal behavior in adolescents and young adults: Systematic review and meta-analysis of longitudinal studies. Int. J. Public Health.

[103] Hom M.A., Joiner T.E., Bernert R.A. (2016). Limitations of a single-item assessment of suicide attempt history: Implications for standardized suicide risk assessment. Psychol. Assess..

[104] Millner A.J., Lee M.D., Nock M.K. (2015). Single-item measurement of suicidal behaviors: Validity and consequences of misclassification. PLoS ONE.

[105] Andreotti E.T., Ipuchima J.R., Cazella S.C., Beria P., Bortoncello C.F., Silveira R.C., Ferrão Y.A. (2020). Instruments to assess suicide risk: A systematic review. Trends Psychiatry Psychother..

[106] Wolgast M. (2014). What does the Acceptance and Action Questionnaire (AAQ-II) really measure?. Behav. Ther..

[107] Tyndall I., Waldeck D., Pancani L., Whelan R., Roche B., Dawson D. (2019). The acceptance and action questionnaire-II (AAQ-II) as a measure of experiential avoidance: Concerns over discriminant validity. J. Context. Behav. Sci..

[108] Rolffs J.L., Rogge R.D., Wilson K.G. (2016). Disentangling components of flexibility via the hexaflex model development and validation of the Multidimensional Psychological Flexibility Inventory (MPFI). Assessment.

[109] Angelakis I., Gooding P. (2021). Experiential avoidance in non-suicidal self-injury and suicide experiences: A systematic review and meta-analysis. Suicide Life Threat. Behav..

[110] Joe S., Baser R., Breeden G., Neighbors H.W., Jackson S. (2006). Prevalence of and risk factors for lifetime suicide attempts among Blacks in the United States. JAMA.

[111] Veresova M., Lamblin M., Robinson J., McKay S. (2024). A systematic review and narrative synthesis of prevalence rates, risk and protective factors for suicidal behavior in international students. Front. Psychiatry.

[112] Ramchand R., Schuler M.S., Schoenbaum M., Colpe L., Ayer L. (2022). Suicidality among sexual minority adults: Gender, age, and race/ethnicity differences. Am. J. Prev. Med..

[113] Cyrus K. (2017). Multiple minorities as multiply marginalized: Applying the minority stress theory to LGBTQ people of color. J. Gay Lesbian Ment. Health.

[114] Staal M.A., Bolton A., Yaroush R., Bourne L., Lukey B.J., Tepe V. (2008). Cognitive performance and resilience to stress. Biobehavioral Resilience to Stress.

[115] Seo E.H., Yang H.J., Kim S.G., Yoon H.J. (2022). Ego-resiliency moderates the risk of depression and social anxiety symptoms on suicidal ideation in medical students. Ann. Gen. Psychiatry.

